# Historical Notice Concerning Aphasia

**Published:** 1875-12

**Authors:** M. Jastrowitz

**Affiliations:** Berlin


### HISTORICAL NOTICE CONCERNING APHASIA.

By DR. M. JASTROWITZ, of Berlin.

(Translated from the "Berliner Klin. Wochenschrift." No. 23, June 7, 1875, by Dr. James I. Tucker.)

Trousseau asserts that the complex symptom, known as aphasia, was first mentioned by Lordat in 1820. We are indebted to Bouillaud for first pointing out its precise anatomical seat by means of a disease of the anterior lobes of the hemispheres which he observed in 1825, and also for an exact formulizing of the idea. Prior to this time, it would seem that aphasia was not defined, as it is at present, to be the inability to form a word *mentally*, the functions of the organs of articulation being at the time unimpaired. Even Abercrombie, who has sometimes been referred to in this connection (although his work on brain diseases did not appear until 1827), was not altogether lucid upon the distinction which exists between a defect in the power of articulation and that of forming the word in the mind, as one may learn by referring to the best in his record of cases bearing upon the subject, (111, 117, 112, 119). Hence it is intensely interesting to know that in the last century, another writer, possessing a nice sense of this signal difference, describes a case of aphasia with hemiplegia incidentally yet with the utmost clearness. This writer was no less than Goethe.

In Chap. 6, Book VII, of "Wilhelm Meister's Apprenticeship," completed in 1797, appears this remarkable passage:

"But alas! this pleasing state was not of long continuance; altogether unexpectedly my father had a shock of palsy; it lamed his right side and deprived him of the proper use of speech. We had to guess at everything that he required; for he never could pronounce the word that he intended. There were times when this was dreadfully afflicting to us; he would require expressly to be left alone with me; with earnest gestures he would signify that every one should go away; and when we saw ourselves alone, he would not speak the word he meant. His impatience mounted to the highest pitch; his situation touched me to the inmost heart. This much seemed certain; he had something he wished to tell me, which especially concerned my interest. What longing did I feel to know it! At other times I could discover all things in his eyes; but now it was in vain. Even his eyes no longer spoke. Only this was clear; he wanted nothing; he desired nothing; he was striving to discover something to me, which unhappily I did not learn. His malady revisited him; he grew entirely inactive, incapable of motion, and a short time afterwards he died."

More briefly, but just as unmistakably, was aphasia noticed at a still earlier period by Van Swieten. *Commentaria in Boerhav. aphorismos*, 1755, "*On Apoplexy*," § 1018. "*Vidi plures, qui ab apoplexia curati, omnibus functionibus cerebri recte valebant, nisi quod deesset hoc unicum, quod non possent vera rebus designandis vocabula invenire; manibus, pedibus, totius corporis nixu conabantur explicare miseri quod vellent, nec poterant tamen. Malum illud per plures annos, saepe*

*insanabilis, perstat.*" (I have known many persons who, after being cured of apoplexy, were able to exercise the brain in all its functions save that this one thing alone was wanting, they could not find the right words for designating things. With their hands, their feet, with an effort of the whole body, these unhappy persons would struggle to make known what they wished, yet were not able. This derangement remained through many years, and was often incurable.)

It is surprising that he regards the right-side hemiplegia which usually accompanies the mental phenomena differently from Goethe, inasmuch as he appends the few lines from Malpighi to the effect that he (Malpighi) after a recurrence of a palsy of the right side resulting from apoplexy, nevertheless "*magnum in memoria et ratiocinio laesionem habuit et quavis minima de causa lacrymabatur.*" (Suffered serious injury in his memory and his reason, and from the slightest cause burst into tears.)

At least the remark of Trousseau does not apply to Van Swieten, viz., that he chose the term aphasia rather than alalia, because it enabled him to draw a sharp line of distinction between this complex symptom and the alalia of the earlier authorities.

"The forms of alalia, of which Sauvages, the two Franks, Bullen, etc., speak, are in the writings of these authors nothing but a conglomeration of contradictory phenomena, which we have of late wrongly endeavored to restore to their former position of honor."—*Clinique Médicale, Tom II.* 669.

